# The International Decimal System of Weights and Measures

**Published:** 1878-11

**Authors:** 


### THE INTERNATIONAL DECIMAL SYSTEM OF WEIGHTS AND MEASURES.

Wepredict that the decennial convention of 1880, for revision of the United States Pharmacopœia, will adopt this system as the American standard. In view of this fact it is certainly advisable to become familiar with the system at our leisure. Aside from any necessity on this account, convenience demands the adoption of this easy and rational standard. We already use it in federal money, and find such decided advantages over that of sterling computation of money, that every one must see at once the benefit of a change from the troy ounce, and its divisions into drachms, scruples and grains to the gram and decimals above and below that unit. In practice it is equally simple and easy as the dollar unit, with decidollar, or dime, and centidollar, or cent.

All that is necessary for the apothecary to buy in order to fill prescriptions written on this system, is a set of gram weights and a gram measure about the size of our present two-ounce graduate measure. The scales now used will, of course, answer for weighing grams as well as grains.

The only trouble in the whole matter arises in the want of experience in the relative value of the gram in grains. In order to overcome this difficulty very soon, it is only necessary to keep in view the number of grains that correspond to the unit or gram weight, the number of inches in a meter and the number of pints in a liter, then the decimals of these units will soon become familiar, so that any amount which we know in grains, inches, minims or fluidounces, can be readily written in grams, liters or meters.

For the convenience of the apothecary, the system is still further simplified. The term gram is used for the weight of solids and measure of liquids. By some writers the term cubic centimeter is used in place of gram for the measure of liquids, because one cubic centimeter (a little more than one-third of an inch square) of water at 60° weighs fifteen grains; but doubtless the term gram will be generally adopted. Furthermore, weights and measures will be generally reckoned by the unit and hundredths. Instead of writing or saying one, two or three decigrams, we say ten, twenty or thirty centigrams, as we say ten, twenty or thirty cents in counting money.

As will be seen in the table below, about fifteen grains make the equivalent of a gram weight, and fifteen drops or minims make the equivalent of a gram measure. Four grams, therefore, by weight and measure, correspond pretty nearly with our present drachm and fluidrachm. If we wish to prescribe about sixty grains or a troy drachm of a solid article, we write, 4 gm. If sixty drops or a fluidrachm of liquid is desired, it may be expressed by the same symbol, 4 gm. The solid will be made to balance a four-gram weight, and the liquid will be made to fill the graduate measure to the line marked 4 grams.

The only difference in counting federal money and metric weights and measures is, that in the former the symbol is placed before the number, thus—$2.50. In the latter the symbol follows, thus—2.50 gm.

| | | |
|---|---|---|
| ℞.—Pulv. opii, | . . . | 6 cg, or 0.06 gm=gr.j. |
| Blue mass, | . . . | 30 cg, or 0.30 gm=gr.v. |
| Tannin, | . . . | 24 cg, or 0.24 gm=gr.jv. |

M. and make four pills.

℞.—Spt. nitrous ether, . . . . 16 gm=f℥jv.

Syrup squills, . . . . . 8 gm=f℥ij.

Paregoric . . . . . . 32 gm=f℥j.

Mix.

TABLE OF INTERNATIONAL WEIGHTS AND MEASURES FOR APOTHECARIES.

| | | | |
|---|---|---|---|
| 10 miligrams (mg), | 1 centigram (cg)=gr. | 0.15=gtt. | 0.15 |
| 10 centigrams, | 1 decigram (dg)=gr. | 1.54=gtt. | 1.54 |
| 10 decigrams, | 1 GRAM (gm)=gr. | 15.43=gtt. | 15.43 |
| 10 grams, | 1 dekagram (Dg)= ʒ | 2.57= fʒ | 2.57 |
| 10 dekagrams, | 1 hectogram (Hg)= ℥ | 3.21= f℥ | 3.21 |
| 10 hectograms, | 1 kilogram (Kg)= ℥ | 32.14= f℥ | 32.14 |

